# Strengthening the Workforce for Equity-Centered Learning Health Systems: Reflections on Embedded Research and Research Generalism: Comment on "Early Career Outcomes of Embedded Research Fellows: An Analysis of the Health System Impact Fellowship Program"

**DOI:** 10.34172/ijhpm.8611

**Published:** 2024-08-19

**Authors:** Brianne Wood, Roya Daneshmand

**Affiliations:** ^1^Thunder Bay Regional Health Research Institute, Thunder Bay, ON, Canada; ^2^Dr. Gilles Arcand Centre for Health Equity, NOSM University, Thunder Bay, ON, Canada

**Keywords:** Embedded Research, Learning Health Systems, Northern Ontario, Health Equity

## Abstract

As embedded researchers in Northern Ontario, Canada, we offer our reflections on Kasaai and colleagues’ 2023 "Early Career Outcomes of Embedded Research Fellows: An Analysis of the Health System Impact Fellowship Program." In our commentary, we draw on our experiences and what is known about embedded research training to examine how to build and strengthen the workforce for equity-centered learning health systems. Does our narrow understanding of outcomes and impacts of embedded research training in Canada affect who benefits and which systems can realize the potential of learning health systems? We identify three areas for deeper analysis: outcomes and impacts at the individual, partnership, and system level, knowledge on the social identities and needs of individuals in embedded research partnerships, and research generalism as a complement to embedded research. Our recommendations suggest tailored approaches to strengthen the workforce capacity for equity-centered learning health systems in Canada.

## Introduction

 The Health System Impact (HSI) Fellowship program, established by the Canadian Institutes of Health Research (CIHR) in 2017 in response to the pan-Canadian Training Modernization Strategy, represents a significant initiative in modernizing doctoral and postdoctoral training within the healthcare sector. As Kasaai et al describe in their recent paper,^1^ the HSI fellowship aims to create value for doctoral students and graduates, health system and academic institutions. In particular, the program aims to strengthen the fellows’ skillsets to include leadership and partnered research competencies, while also adding value to health system institutions by improving their use of research, and academic institutions that can leverage the next generation of researchers who have an impact-oriented skillset. The HSI Fellowship seeks to modernize doctoral and postdoctoral training by embedding individuals in health organizations, enabling them to lead impact-oriented projects by applying their research skills.

 There are several reports asserting early successes of the HSI Fellowship, including positive perceptions of the program’s value and increases in applicants and potential host organizations. Some authors suggest that these findings reflect the progress of learning health systems in Canada.^2,3^ A recent synthesis of frameworks explicitly presents embedded research as the manifestation of learning health systems, and articulates the types of research evidence that mobilize each learning gear.^4^ Other authors map out how different embedded research competencies are prioritized and implemented at different scales of health system learning.^2^ By financially supporting partnered projects for PhD trainees, postdoctoral fellows, and now early career researchers,^5^ the CIHR HSI Fellowship is increasing the visibility and appeal of embedded research, though it is still unclear how these embedded researchers, their projects, and their partnerships are translating into learning health systems.

 As embedded researchers and learning health system scientists looking to build and sustain equity-centered learning health systems in Northern Ontario, Canada,^6^ we aim to understand, adapt, and then apply evidence to build embedded research capacity, specific to our context. Lessons from CIHR’s HSI Fellowship could provide practical and context-specific guidance to build and sustain equity-centered learning health systems. Specifically, there is a need to understand the relationships between the trainee and their mentors, the characteristics of “embeddedness”^7^ of the researcher, their partnerships and system, the project, and the respective outcomes and impacts. However, there are conceptual and empirical knowledge gaps around training embedded researchers as an implementation strategy for equity-centered learning health systems, let alone the outcomes that such a system intends to achieve.^4^ Reflecting on Kasaai and colleagues’^1^ report of the career trajectories of CIHR’s HSI fellows, we ask:

1. What are the outcomes and impacts of embedded researcher training and how do these contribute to equity-centered learning health systems?
2. How equitable is embedded researcher training, for the trainees and partnership members (eg, mentors)?
3. What is known about “generalist researchers” and in what ways can embedded research training support their development?

###  1. Career Trajectories – Part of the Picture, Not the Whole Picture

 Kasaai et al^1^ used established methods to understand employment statuses of previous HSI postdoctoral fellows. The authors noted that descriptive methodologies were insufficient to draw causal conclusions, although the diversity of roles and feelings of career preparedness indicated the potential for learning health systems. This work highlights an important—and testable—assumption: that enhancing the embedded research workforce will lead to a learning health system. This descriptive analysis contributes new and important knowledge about learning health system workforce strategies, while also highlighting the significant unknowns that further research should investigate. For example, learning health system scientists could consider: in-depth examinations of job responsibilities, team relationships, titles, places, and change over time to understand the composition of the embedded research workforce; describing and analyzing variability among embedded research models^7^ and associated outcomes; mapping individual researchers, their teams, and projects to specific learning gears^4^; and, identifying the ways that system learning was embodied. These descriptions will provide practical insight for leaders who are trying to structure, resource, and sustain learning health systems in different contexts and can also support trainee and early career embedded researchers in understanding different career pathways.

 To advance the science of equity-centered learning health systems and potential workforce development strategies, we also need to move beyond descriptive accounts of embedded research to understand the outcomes and impacts of embedded researchers, their projects, and partnerships over time. We support earlier recommendations for multi-dimensional evaluation of the CIHR HSI Fellowship,^8^ recognizing the need and value of rigorously measuring and qualifying the outcomes and impacts of embedded research, co-produced research, and equity-centered learning health systems.^4,9^ Not only will this type of evaluation provide important information for decisions related to CIHR’s HSI Fellowship, these analyses could also facilitate systematic assessment of health research partnerships,^9^ embedded research,^7^ and equity-centered learning health system outcomes.^4^

###  2. Equity-Centered Training for Equity-Centered Learning Health Systems

 The equity-centered learning health system workforce must define what equity means in their context, then systematically and thoughtfully collect and use relevant data in their learning.^4,6^ The workforce must also means analyze what and whose knowledge and knowledge systems are prioritized and valued, and intentionally including diverse perspectives and discourses, is important.^10^ Subsequently, training pathways for this workforce should also reflect these key principles.

 Kasaai and colleagues^1^ leveraged limited demographic data to conduct sex-stratified analysis of their findings, ultimately concluding that few sex-specific differences were observed among the career trajectories of HSI fellows. Perhaps unsurprisingly, this report confirms other analyses that female PhD graduates are less likely to end up in academia or private sector than their counterparts, though lacks the data to examine correlations by any other identity factors. Kasaai and colleagues, among others, have called for deeper investigations into career outcomes according to identity factors, including gender, race, and geography for example. The recent emphasis on equity, diversity, and inclusion within health research eco-systems, such as Canada’s Gender Based Analysis Plus, requires that research authors critically analyze how sex, gender and other identity categories contribute to inequities, which requires collecting, analyzing, reporting, and acting on corresponding data. Using the HSI Fellowship as an example, inequities might be observed in who is awarded a fellowship, the types of projects that are most highly rated, the places and systems in which HSI fellows are placed, the supports that are provided to the fellows, and of course, existing systemic challenges external to (but intersecting with) the institutions in which the fellowship operates. These inequities are also likely for embedded research trainees outside of the fellowship. Inequities in experiences and outcomes for embedded researchers may have tangible impacts on their career trajectories and the impacts that they want to make. Without explicitly examining and addressing the inequities in who and how we train embedded researchers, we will perpetuate inequities in academic and health system institutions, as well as the equity-centered learning health systems we are trying to create and nurture.

 The HSI Fellowship is well-positioned to support embedded research trainees who face systemic barriers because the “experiential” aspect of the fellowship relies on mentorship from both health system and academic mentors.^11^ Mentors from the health system and academia—leaders in the HSI Fellowship—play important roles in trainees’ abilities to create and sustain relationships and build capacity in relevant learning health system competencies. Mentorship is an often-used strategy^12^ to support trainee and early career researchers, particularly individuals from equity-deserving communities. However, diverse academic and health system leaders might avoid mentoring embedded researchers because of the significant additional mental and emotional workload that they already endure or might be subjected to. To attract, nurture, and sustain mentors in equity-centered learning health systems, there must be dedicated supports and clear value—short- and long-term—for those who take on these roles, individually, at the partnership-level, and their organization/system and community. As mentioned, robust evaluation approaches that include the needs of mentors, their approaches to mentorship, and apply an intersectional lens is necessary to inform capacity-building for equity-centered learning health systems.

###  3. Research Generalism – Team- and Place-Based Learning

 Individual characteristics of embedded research trainees and their mentors will influence training experiences, outcomes, and impacts, and likely, the potential to building equity-centered learning health systems. The Canadian Health Services Policy and Research Alliance’s enriched core competencies framework, updated May 2024,^13^ and interviews with early career researchers,^5^ highlight the important skill sets that embedded research learning opportunities like the HSI Fellowship can target. Even more than before, this new competency framework emphasizes the significance of skills beyond the traditional research methodologies and content areas, such as leadership, negotiation, and understanding health systems and organizational context. Petrie et al^2^ frame the CIHR’s HSI fellows, perhaps embedded researchers generally, as “general contractors” who must demonstrate a breadth of skills, in addition to deeper knowledge in project-specific areas. We feel that positioning embedded researchers as research generalists aligns with our experiences as embedded researchers building equity-centered learning health systems. Our “embeddedness”^7^ involves being hosted by academic institutions (a research institute and medical university) though typically remotely working due to the vast geography of our region. We spend most of our time on research facilitation and knowledge translation with multiple Northern Ontario health system and community partners, helping to answer questions identified by these partners. At this point, a smaller portion of our time focuses on “research production” related to assessing equity-centered learning health system progress locally.

 We also acknowledge that research generalism is not new, though perhaps contrary to how academic training is often conceptualized. Generalism, particularly clinical generalism, is the norm in rural, remote, and isolated communities where clinicians, researchers, and health system decision-makers have no choice but to collectively build and transform their systems, given low resources, heterogeneous communities and places.^6,14,15^ Research generalism can better align with knowledge systems that rely on “whole” ways of learning and honour forms of evidence beyond traditional research outputs.^10,15^

 The shift toward training research generalism is reflected in CIHR’s modernized competency framework.^13^ Are our systems ready to support and sustain these generalist experts, and is it feasible that we develop research generalists outside of traditional doctoral programs and national fellowships? Ultimately, generalist researchers are experts in their local contexts and “places,”^6,14^ while also navigating between scales of learning and leveraging their specialized research skills across multiple domains.^2,13^ This generalist expertise requires active participation in local communities to understand their needs and preferences (health and otherwise), building partnerships within and between local health and academic organizations, understanding care delivery, working within existing infrastructure, among. Importantly, embedded researchers—those who are affiliated with both academia and policy/practice organizations—are not necessarily research generalists, and vice versa. Both roles are important to advance learning health systems, although the latter is less understood and likely, less valued. Generalism challenges traditional assumptions and practices around who can be considered an “expert,” approaches to curriculum and teaching, resourcing for training and careers, and may require different mentorship styles, new milestones, and opportunities to make impacts. When and how learning health system institutions prioritize places and communities (ie, generalism) over themes and content areas will impact what individuals, partnerships, and systems are most likely to realize the benefits of equity-centered learning health systems.

## Recommendations

 Given the relational nature of mentorship,^11^ equity-centered system work,^6^ and system learning,^10^ there is no one-size-fits-all approach to training embedded researchers just as there is no single best way to build and sustain equity-centered learning health systems. Kasaai and colleagues’ report on the early career outcomes of CIHR’s HSI fellows^1^ contributed to the body of knowledge related to building an embedded research workforce, in which the HSI fellowship plays a significant role in Canada. In Table, we summarize the challenges we identified and potential opportunities to strengthen training and workforce development for equity-centered learning health systems.

**Table:** Current Challenges, Potential Solutions and Accountable Parties in Developing Capacity for Equity-Centered Learning Health systems

**Identified Challenges**	**Potential Solutions**	**Who to Lead**
Existing career trajectories and perceived competencies analyses do not provide insight into the nature, extent, outcomes, and impacts of embedded research, within the HSI Fellowship or local-level learning health systems	Systematically evaluate embedded research training and health research partnerships over time, using validated tools	- Researchers (eg, learning health system scientists) - Health system and research funders
Lack of data, analysis, and interpretation on social identify data of embedded research trainees, their mentors, and their team members	Collect social identity data in a safe and secure way and commit to using the information to improve how embedded research training is delivered	- Learning health system institutions (health system, and research organizations) - Academic institutions^a^
Heterogeneous approaches to mentorship in embedded research and unclear value for mentors	- Develop mentor-specific resources to support their participation - Determine and communicate the value for health system and academic mentors to participate	- Health system and research funders - Learning health system institutions (health system and research organizations) - Academic institutions^a^ - Researchers (eg, learning health system scientists)
Research generalists are necessary to advance equity-centered learning health systems, but current systems are not set up to sustain this work	- Acknowledge that research generalism is its own specialty, and might overlap (though not necessarily) with embedded research - Shift from thematic funding, one-time opportunities to sustained place-based resourcing	- Learning health system institutions (health system and research organizations) - Academic institutions^a^ - Health system and research funders

Abbreviation: HSI, Health System Impact. ^a^ In this table, we denote “academic institutions” as distinct from learning health system institutions (eg, research organizations), though in some cases, they might be the same. By identifying academic institutions separately, we acknowledge the roles of people and institutions with additional accountabilities and mandates, including education and service.

## Conclusion

 To build a workforce who will advance equity-centered learning health systems in Canada, we recommend a deeper analysis of embedded researchers, their projects and partnerships, and the respective outcomes and impacts. We examine how research generalism can complement embedded research training, and make unique, important contributions for equity-centered learning health systems. To realize the potential of equity-centered learning health systems and to improve health and care outcomes, our training pathways must build capacity in embedded research and research generalism, consider new ways to structure and resource this work across career trajectories and systems, and undergo rigorous evaluation.

## Ethical issues

 Not applicable.

## Competing interests

 Authors declare that they have no competing interests.
